# Tax-Free Benefactions

**Published:** 1920-07-10

**Authors:** 


### TAX-FREE BENEFACTIONS.

If everyone were to give the Biblical tenth of income to charity, and were allowed by the Chancellor of the Exchequer to deduct that tenth from income, paying tax on the balance, there is no doubt that the effect upon the gross revenue received by the Treasury would be very considerable, and would necessitate additional direct or indirect taxation, either by a higher rate of income tax or by other means.

But, as has been suggested by Sir Theodore Morison and Professor Adami, in an interesting correspondence in *The Times*, when such deductions from national income save equal or greater additions to national expenditure, the net result to the State is that the process produces something which is as broad as it is long, and, to use an Irishism, possibly a little broader.

So much depends, in the consideration of this matter, on what may, for the purposes of the transaction, be defined as private beneficence. Sir Theodore Morison and Professor G. Adami very naturally suggest universities as coming within a scheme of tax-free gifts, the latter also includes hospitals, and, we, of course, are inclined to applaud. But there are others who might in their turn bring forward the claims of the benefactor who deals with blind institutions, orphanages, rescue work or private burdens, such as the support of indigent relations and superannuated servants. All might argue, with more or less truth, that they are relieving the State of possible expense.

### In the States.

The Chancellor of the Exchequer has heard of this question of tax-free benefactions before, indeed, it was considered and pushed on one side by the Royal Commission on Income Tax, who were unable to recommend that an allowance which is made in the United States should be made in the United Kingdom. In the States the arrangement goes further than the tithe of Scripture; a deduction is allowed from the taxable income of an individual of contributions or gifts to corporations organised or operated exclusively for religious, charitable, scientific, or educational purposes, or for the prevention of cruelty to children or animals, to an amount not exceeding 15 per cent. of the taxpayer's net income as computed without the benefit of this provision. No deduction is allowed in computing the taxable income of a company or corporation.

Here, to the very small extent that the system prevails, the arrangement is the other way about. The corporation or company, or, for the matter of that, business firm or individual, can, in certain circumstances, benefit in the matter of income tax by a benefaction. But this benefaction must be in the shape of a gift, classed as a trade expense, to a hospital or other local institution which is helpful in the matter of treating employees or by analogous service. It needs but little imagination to picture how such a gift might, in certain circumstances, mean escape from excess profits duty, the effect being that the local hospital receives a full pound.

That, however, is but an unimportant side issue. The main question is what would such a concession cost the Government and, apart from any sentimental motive, what value would the Government get in return for the tax foregone? The first part of this question is exceedingly hard to answer. The total revenue, we suppose, of what may be described as regular and recognised charities of the United Kingdom (which in any State scheme would surely also have to be registered) is about £20,000,000. Of that sum a large part, from legacies, is subject to special and separate taxation indistinguishable in amount because it often represents estate duty on a sliding scale as well as legacy duty. On the other hand, the revenue from invested funds is tax-free. Another large part, unknown in amount, is made up of contributions on the part of patients and inmates of hospitals and institutions. Of the balance, pure charitable donations, much is represented by the small contributions of persons below the income-tax limit, either by direct gifts, by casual offerings to alms-boxes or through Hospital Sunday, or by periodical agreed deductions from wages for local institutions. Let us assume that our income-tax payer gives £10,000,000 a year to institutions such as are above described in the Statute of the United States. Let us put the tax allowable at a flat rate of 4s. 6d. in the pound, half-way between the 3s. rate on smaller incomes, and the 6s. rate on larger. The hen-roost of the Treasury is robbed to the extent of £2,250,000. But we have not dealt with the advocates of private benefactions to Universities and other educational forms of voluntary effort, the gross extent of whose gifts is, so far as we are aware, not to be ascertained without considerable research. Let us assume that the incidence would reach another £750,000. That would mean that the Government would have to provide (or rather to do without) three millions of money and, as one cannot expect pure altruism of the Treasury, to forgo this amount in order to save voluntary effort from shipwreck would be in order to spare the public purse the costly work of replacement by State organisation. At first sight it seems worth it.

### Reflections.

But, on reflection, surges over one an unexpected wave of sympathy for the Inland Revenue authorities. Just at the moment when a clever system of assessment and calculation has been instituted comes the suggestion of a system of rebates which would mean enormous calculation, supervision, and an intricate system of safeguards against chicanery. Just picture the income-tax return of the person who, as many men and women do, makes a practice of giving to institutions. Think of the unfortunate inspector going through the wad of receipts, examining the dates, the amounts, and ascertaining that the charity has been registered at the appropriate Government registry. Consider the extra work of the institutional secretary in obtaining and retaining, through the filling-up of many forms and the presentation of certified accounts in required style, registration. The spectacle is sombre and, to the wavering enthusiast of this idea, deterrent.

Moreover, there is an element of humbug in the suggestion, if we may use the word in an offensive sense.

We are much more in sympathy when it comes to a question of expenditure. State aid does not necessarily mean State interference to an extent that matters. We agree that it is the universal experience that departmental administration is far more costly and less effective than is private control. This is very true, but the Government has its hands far too full to take the universities and hospitals from the control of those who can be trusted to govern them with wisdom and economy.

